# A retrospective study of the Dual-channels Bolus Contrast Injection (Dc-BCI) technique during endovascular mechanical thrombectomy in the management of acute ischemic stroke due to large-vessel occlusion: a technical report

**DOI:** 10.3389/fneur.2025.1508976

**Published:** 2025-02-18

**Authors:** Ying Jiang, Yi-Lin Liu, Xiang Zhou, Qin-Qin Shu, Lan Dong, Zheng Xu, Jie-Qing Wan

**Affiliations:** ^1^Department of Neurosurgery, Cerebrovascular Diseases Center, Renji Hospital, Shanghai, China; ^2^Department of Nursing, Changzheng Hospital of Naval Medicine University, Shanghai, China; ^3^Department of Neurosurgery, Quzhou Hospital of Traditional Chinese Medicine, Quzhou, Zhejiang, China; ^4^Shanghai No. 4 People’s Hospital Affiliated to Shanghai Tongji University School of Medicine, Shanghai, China; ^5^Department of Emergency Department, Changzheng Hospital of Naval Medicine University, Shanghai, China

**Keywords:** thrombectomy, stroke, dual-channels bolus contrast injection, thrombus distal end, stent retriever, reperfusion after ischemia

## Abstract

Endovascular mechanical thrombectomy (EMT) is an effective treatment for acute ischemic stroke and identifying the precise thrombus size remains key to a successful EMT. However, no imaging modality has been able to provide this information simultaneously and efficiently in an emergency setting. The present study introduces a novel technique named dual-channel bolus contrast injection (Dc-BCI) for determining thrombus size and location during EMT. In the *in vitro* study, the Dc-BCI demonstrated an accurate projection of the thrombus size, as the actual thrombus diameter (R^2^ = 0.92, *p* < 0.01) and length (R^2^ = 0.94, *p* < 0.01) exhibited a high degree of correlation with that of obtained from Dc-BCI. Consequently, between February 2023 and August 2024, 87 patients diagnosed with acute cerebral large vessel occlusions were enrolled in the study and received EMT for the treatment of acute cerebral large vessel occlusions. The Dc-BCI was successfully performed in all patients to measure the diameter and length of the thrombus. These information were used to select an appropriate stent-retriever for EMT. The restoration of blood flow was achieved in 84 patients (96.6%) to an mTICI score of 2b/3. Additionally, a low incidence of postoperative complications was observed (e.g., subarachnoid hemorrhage 8% and cerebral hemorrhage 5.7%). In conclusion, it can be posited that the Dc-BCI has the potential to enhance the outcomes of EMT, as it is capable of revealing the thrombus size information, which optimizes the interaction between the stent retriever and the thrombus, while simultaneously reducing the risk of vascular injury that is associated with the prolonged use of the stent retriever.

## 1 Introduction

Cerebral occlusion is a devastating clinical event that leads to an acute ischemic stroke and often results in a severe neurological deficit or death. Several randomized clinical trials have shown that endovascular mechanical thrombectomy (EMT) is an effective treatment for acute ischemic stroke (1–4). A subsequent study, however, has demonstrated that up to half of stroke patients have an unfavorable outcome, mainly due to unsuccessful recanalization (5). It is currently understood that the success of EMT is contingent upon a multitude of variables, which can be classified into three primary categories: interventional technique- (6, 7), stent-retriever- (8, 9), and thrombus-related factors (10–14). Among these variables, the size and location of the thrombus have been identified as crucial prognostic markers (10–14). It is therefore essential to conduct a detailed and accurate assessment of the size and location of the thrombus in order to maximize the interaction between the stent retriever and the thrombus (15), while simultaneously reducing the risk of injury to the blood vessel caused by the stent retriever (16–18). It is anticipated that this will result in a reduction in the number of attempts and difficulty of EMT, as well as a reduction in the risk of thrombus fragment embolization in a new vascular territory.

Although it is desirable to provide detailed thrombus characteristics, this may not always be feasible in the context of daily practice. For instance, although contrast-enhanced CT angiography (CTA) has been the primary diagnostic imaging modality for large-vessel occlusion, it is a highly time-consuming process (19). Furthermore, its ability to provide information regarding the thrombus’s length (especially the distal location) is limited due to the absence of distal flow from the collaterals to a major occlusion (20) and other serial variables (21). Additionally, the MR T2- (22, 23) and susceptibility-weighted sequence (24) have also been shown to provide vital information regarding the thrombi location and length. However, emergent MR is not widely used due to limited availability and the presence of comorbidities in patients. Since misinterpreting the thrombus size can lead to delayed or failed recanalization, this issue poses a significant challenge to clinicians during EMT. This study introduces a novel technique called dual-channels bolus contrast injection (Dc-BCI) for determining the thrombus size and evaluate its impact on achieving successful reperfusion during EMT.

## 2 Materials and methods

This study was conducted in accordance with the Declaration of Helsinki. The need for informed consent was waived by the institutional ethics committee due to the retrospective nature of the study.

### 2.1 Patient enrollment

This is a retrospective post-hoc analysis of data from patients with acute ischemic stroke who received medical care at our medical center. The inclusion criteria were as follows: (1) diagnosis of acute ischemic stroke due to large vessel occlusion (LVO) in the anterior circulation [involving the internal cerebral artery (ICA), middle cerebral artery (MCA) segment 1 (M1 & M2) and anterior cerebral artery (ACA) segment 1 (A1 & A2)] and the posterior circulation [involving the basilar artery (BA) and the intracranial vertebral arteries segment 4 (V4)]; (2) significant perfusion mismatch between stroke severity and infarct volume; (3) the time limit from the onset of the stroke is less than 24 h; (4) the cut-off score using the National Institutes of Health Stroke Scale (NIHSS) score is set at ≥6; (5) the patient received stent retriever during EMT; and (6) written informed consent was obtained from the patient’s legal guardian prior to treatment. The modified Thrombolysis in Cerebral Infarction score (mTICI) was used to evaluate the results of EMT. A successful recanalization was defined as an mTICI score of 2b to 3.

After enrollment, the patients were further divided into two groups based on the underlying cause of their ischemic stroke: intracranial atherosclerosis (ICAS) and embolism group. The ICAS was defined based on the first-pass effect (25) and the observation of stenosis at the site of occlusion during emergency medical treatment (EMT). The embolism was diagnosed if there was no evidence of focal stenosis after clot retrieval and/or an embolus was retrieved during EMT.

### 2.2 The methodology of the dc-BCI

**Figure 1:**
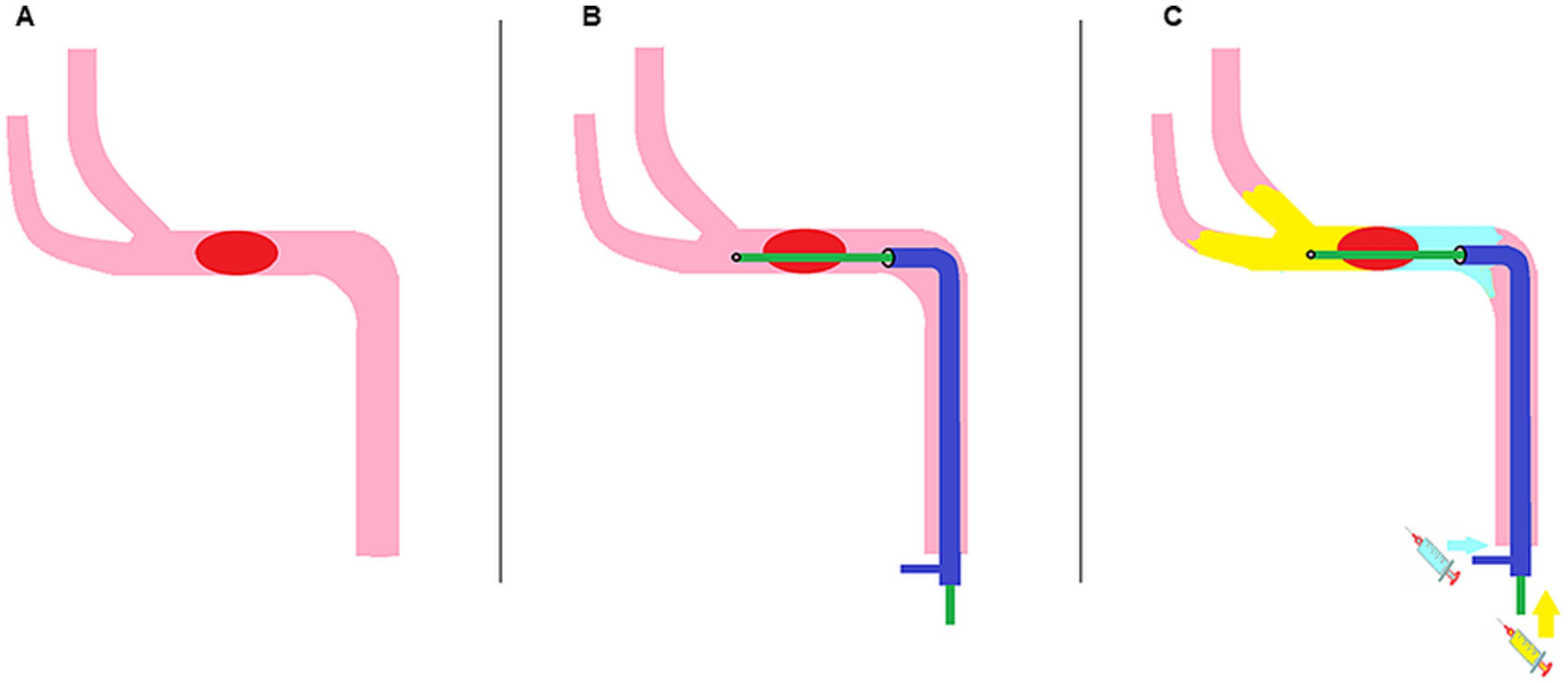
Schematic diagram of the Dc-BCI concept. An acute ischemic stroke occurs when a thrombus obstructs the cerebral anterograde blood supply **(A)**. First, the aspiration catheter (shown in dark blue) was positioned at the proximal end of the clot, while the microcatheter (shown in green) traversed the clot and reached the proximal end of the clot **(B)**. Subsequently, contrast was administered through both the aspiration catheter and the microcatheter in a simultaneous manner **(C)**. The contrast agent from the aspiration catheter (depicted in light blue) will accumulate at the proximal end of the clot. Meanwhile, a portion of the contrast agent injected from the microcatheter (shown in yellow) will reflux to the distal end of the clot. Consequently, the clot is discernible on the angiogram as regions of contrast filling defect. Dc-BCI: dual-channel bolus contrast injection.

The methodology of the DC-BCI is depicted in Figure 1. Firstly, the optimal working angle was identified, and the proximal end of the thrombus was located via contrast injection from the aspiration catheter. Once the proximal occlusion position was identified, a micro-guidewire was employed to facilitate the navigation of a microcatheter into the distal portion of the occluded site (see Figures 1A,B). Subsequently, contrast was injected in unison from both the microcatheter and aspiration catheter (Figure 1C). The contrast injected via the aspiration catheter ceases at the proximal end of the clot, whereas that injected via the microcatheter flows both forward and refluxes to the distal end of the clot. This technique, designated as the DC-BCI, entails the filling of contrast at both the proximal and distal aspects of the thrombus, while leaving the thrombus itself devoid of contrast. During EMT, the thrombus can be distinctly discerned on the angiogram as the region of contrast filling defect. The length of contrast filling-defect could be quantified on the DSA computer, revealing the thrombus’ size, including the length and diameter.

### 2.3 Lab experiment setting

The efficacy and reproducibility of the Dc-BCI were initially assessed *in vitro* using the Endovascular Surgical Simulator IX-01 (EVE; FAIN Biomedical, Okayama, Japan). The systemic arterial model was maintained in a continuous circulation with saline at a temperature of 37°C. An embolic sham thrombus, created as previously described (26), was randomly divided into small pieces, with the size of each piece recorded prior to testing. The sham thrombus was then placed at the MCA M1 segment or BA. The microcatheter surpassed the thrombus while the aspiration catheter was placed proximal to the thrombus. Then, the Dc-BCI technique was performed. Saline colored with red ink was used to mimic the contrast distribution in arterial vessels. The length and diameter of each ink defect were documented, and these values were subsequently integrated with the data pertaining to the artificial thrombi’s length and diameter, respectively.

### 2.4 Protocol of endovascular procedures

The interventional procedures were carried out using the Philips Asurion 7 B20/15 biplane angiography system (Philips, Cambridge, US) under general anesthesia. Firstly, an 8-French (F) femoral sheath was inserted into the patient’s femoral artery. Then, a 90-cm 8F multipurpose (MPA) guide catheter (Cordis®, Santa Clara, CA, USA) was positioned at the opening of the proximal internal carotid artery with the aid of a 5F MPA catheter. The guiding catheter was used to advance the aspiration catheter in a coaxial manner. A three-dimensional (3D) rotational angiography was then performed to obtain information about the cerebral arteries and occlusion. Once the exact proximal occlusion position was identified, the Synchro® microguidewire (Stryker Neurovascular, Fremont, CA, USA) was used to navigate the Rebar-18 Microcatheter (Medtronic, Minneapolis, MN, USA) into the portion distal to the occluded site. The contrast was then introduced via the microcatheter to confirm its accurate positioning within the arterial lumen. Then, the Dc-BCI technique was performed. Upon reaching the true arterial lumen, the microcatheter was slowly withdrawn under a negative roadmap with a continuous slow injection of contrast until a sudden change in contrast volume or arterial lumen diameter was observed, indicating that the microcatheter had reached the distal portion of the occlusion. At this stage of the procedure, the microcatheter was advanced to a slight degree, either with or without the utilization of a microwire. This enabled the microcatheter opening to dislodge from the thrombus and remain within the arterial lumen of the distal thrombus site. Subsequently, the Dc-BCI was performed and the primary surgeon and first assistant simultaneously injected contrast via the microcatheter (1 mL contrast injected by 1 mL syringe) and aspiration catheter (up to 10 mL contrast injected by 20 mL syringe), respectively, under the roadmap or angiographic condition. The contrast injected via the aspiration catheter ceases at the proximal end of the clot, whereas that injected via the microcatheter flows both forward and refluxes to the distal end of the clot. This entails the filling of contrast at both proximal and distal aspects of the thrombus while leaving the thrombus itself devoid of contrast. Consequently, the clot can be distinctly discerned on the angiogram as the regions of contrast filling defect. Subsequently, the length of the region contrast filling-defect was quantified on the DSA computer, revealing the clot’s size including the length and diameter. The proximal and distal diameters of the artery were gauged based on contrast filling regions, thereby informing surgeons of the diameter data pertinent to the stent retriever. Next, a stent retriever [Embotrap™ (CERENOVUS, Johnson & Johnson Medical Devices, Irvine, CA, USA) or Solitaire® (Medtronic, Minneapolis, MN, USA)] of appropriate size was deployed. Five minutes later, the aspiration catheter was maneuvered up to the clot or as close to the proximal of the thrombus as possible and initiated with continuous aspiration. The stent retriever was then slowly withdrawn into the aspiration catheter, which was then removed completely with continuous suction from the aspiration catheter. The aspiration catheter was then totally withdrawn from the femoral sheath. An angiogram was instantly performed to check for any remaining thrombi and the reperfusion status. The Dc-BCI and EMT procedure maybe repeated if necessary. For refractory occlusions caused by atherosclerosis, an intracranial stent may be used. At the end of EMT, the XperCT was performed to visualize cerebral tissue, potential hemorrhage, and contrast agent retention before removing the guiding catheter. The hemostasis in the groin puncture site was achieved with the Angio-seal vascular closure device (St. Jude Medical, Plymouth, MN, USA).

### 2.5 Statistical analysis

The data were presented as mean ± standard deviation. Statistical significance was defined as *p*-values of an alpha level of 0.05 or less. Linear regression is used to quantify the association between Dc-BCI-projected thrombus length and thrombus length as observed in real-time. Prism 9 (GraphPad, Boston, MA, USA) was used for all statistical analyses.

## 3 Results

### 3.1 Lab experimental results

**Figure 2:**
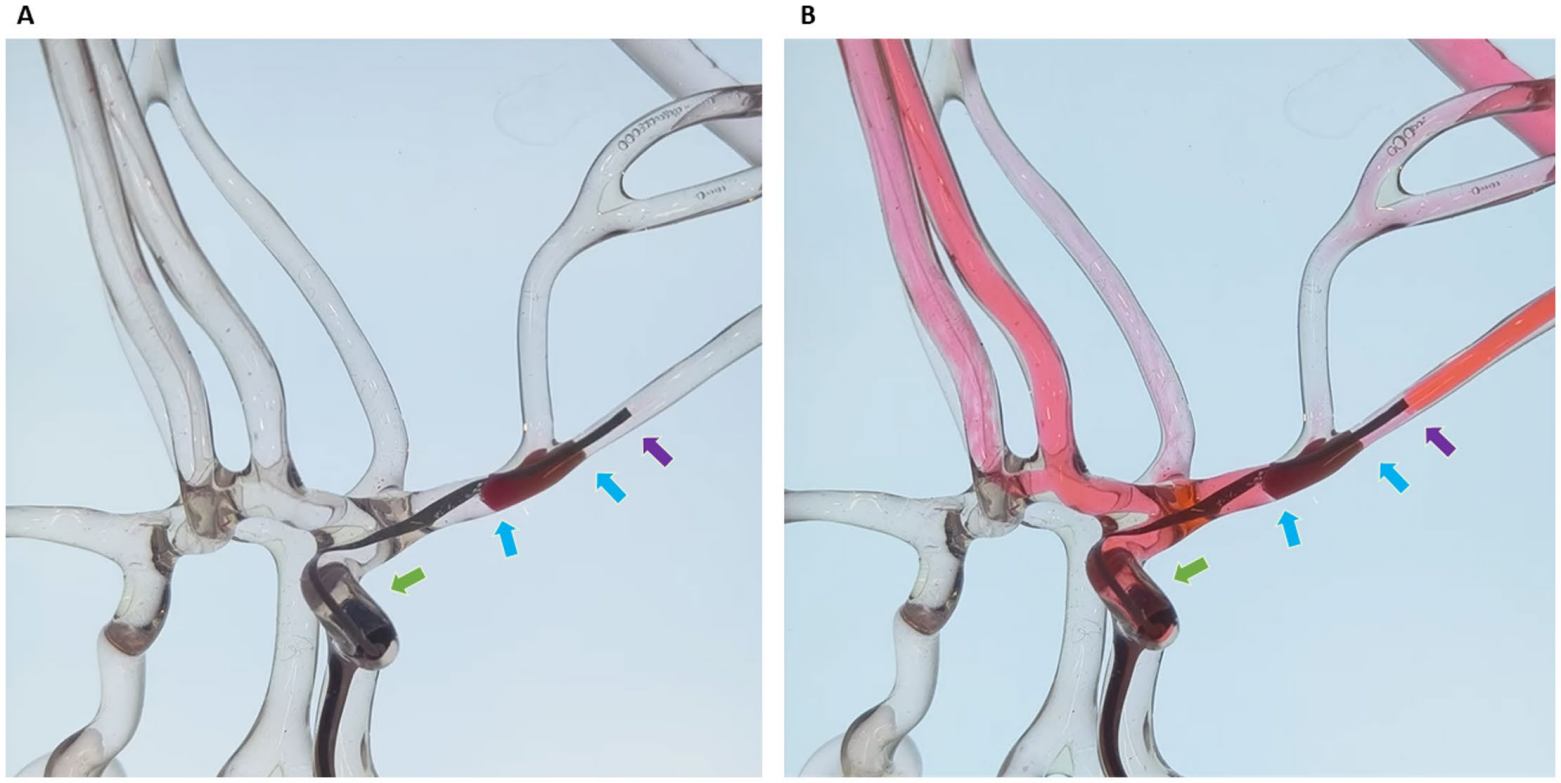
The dual-channel bolus contrast injection (Dc-BCI) technique was evaluated in an *in vitro* setting. A sham thrombus was introduced to occlude the large cerebral arteries in the endovascular surgical simulator **(A)**. The microcatheter traversed the thrombus while the aspiration catheter was positioned proximal to the thrombus. Red-ink-colored saline was used to mimic contrast distribution in arterial vessels. To simulate the distribution of contrast in arterial surgical simulator, red-ink-colored saline was utilized. The Dc-BCI procedures were performed, and the area of ink defect reflected the length and diameter of the sham thrombus **(B)**. *Green arrow: tip of aspiration catheter; Purple arrow: tip of microcatheter; Blue arrows: proximal and distal ends of embolus (also known as total thrombus length).

**Figure 3:**
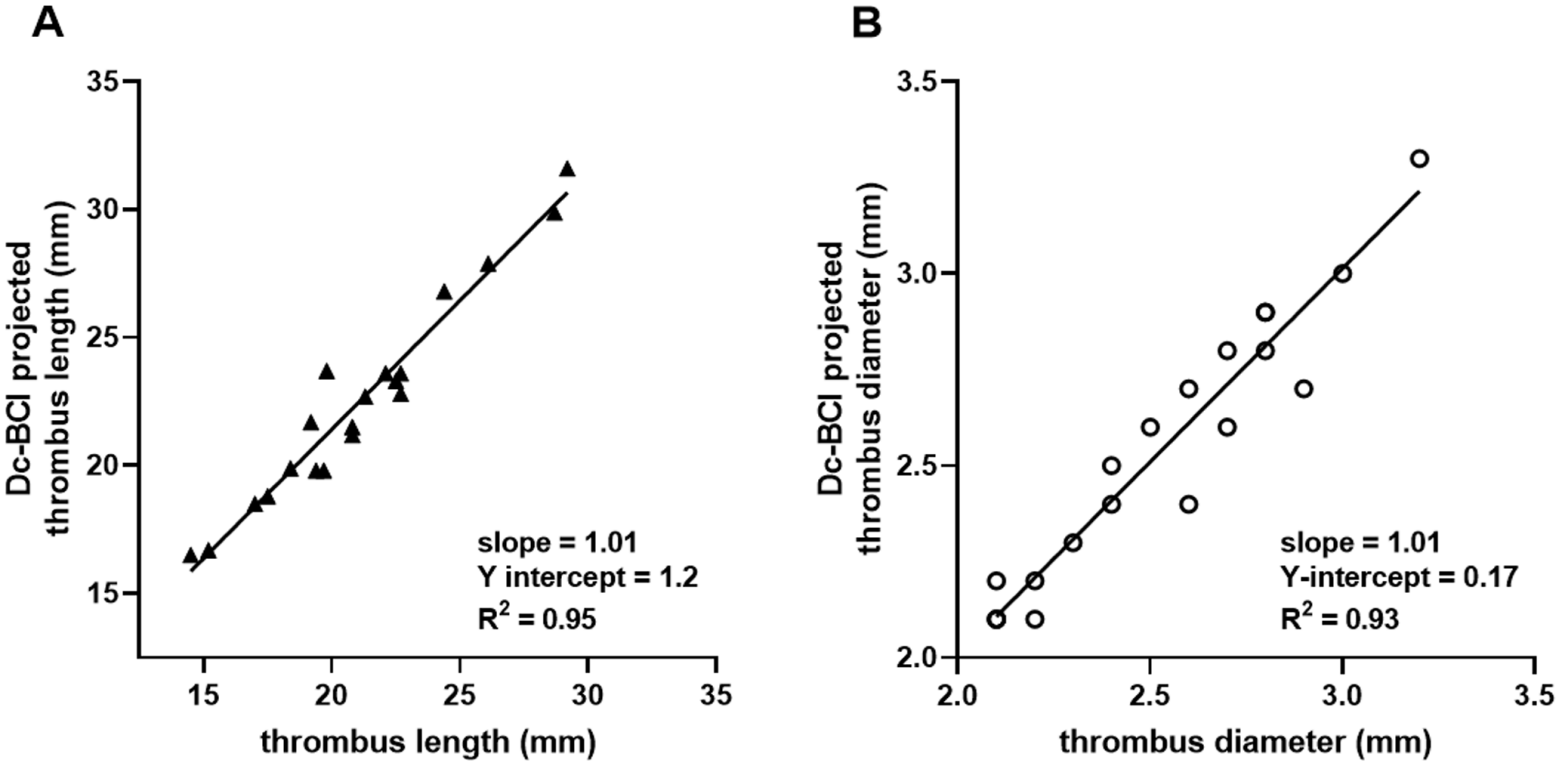
The efficacy of the dual-channel bolus contrast injection (Dc-BCI) technique was analyzed *in vitro*. In a total of 20 trials, the Dc-BCI demonstrated a tight correlation between its projection and both the actual thrombus diameter (R^2^ = 0.92, *p* < 0.01) **(A)** and length (R^2^ = 0.94, *p* < 0.01) **(B)**.

The efficacy of the Dc-BCI was initially validated *in vitro* using a blood vessel model (Figure 2). A total of 20 tests were conducted. The data indicated that the Dc-BCI could provide an accurate projection of the thrombus size, as the actual thrombus diameter (R^2^ = 0.92, *p* < 0.01, Figure 3A) and length (R^2^ = 0.94, *p* < 0.01, Figure 3B) exhibited a high degree of correlation with that of obtained from Dc-BCI. These findings collectively corroborate the efficacy of the Dc-BCI in thrombus size estimation during EMT.

### 3.2 Clinical trial results

Between February 2023 and October 2023, 87 consecutive patients undergoing EMT were recruited for the current trial. Demographic information is summarized in Table 1. Among them, 56 participants were male (64.6%). The mean age of the patients was 62.8 ± 20.2 years. The first-pass effect was observed in 56 patients (64.4%). The Dc-BCI was successfully performed in all patients. The diameter and length of the thrombus were determined based on the region of contrast-absence observed in the DSA imaging. Thereafter, a stent retriever with an appropriate size was selected for the EMT procedure. After EMT, blood flow was restored to an mTICI score of 2b or 3 in 84 patients (96.6%). Postoperative subarachnoid hemorrhage and cerebral hemorrhage were observed in 7 (8%) and 5 (5.7%) patients, respectively.

**Table 1:** The demographic information of participants.

	Demographic information
*n*	87
Age	62.8 ± 20.2
Male (%)	56 (64.6%)
NIHSS score	17.6 ± 7.8
Hypertension	68 (78.2%)
Diabetes mellitus	42 (48.3%)
Current smoker	52 (59.8%)
Stroke subtype (%)	
ICAS	56 (64.4%)
Embolism	31 (35.6%)
Artery occlusion site (%)	
MCA	67 (77%)
ACA	1 (1.1%)
BA	18 (20.7%)
VA (V4)	1 (1.1%)
mTICI (%)	
0–2A	3 (3.4%)
2B–3	84 (96.6%)
Complication	
Asymptomatic ICH	3 (3.4%)
Symptomatic ICH	2 (2.3%)
Vessel dissection	0 (0%)
Arterial perforation	1 (1.1%)
Isolated SAH	7 (8%)

NIHSS, National Institutes of Health Stroke Scale; ICAS, intracranial atherosclerosis; ICA, Internal carotid artery; MCA, middle carotid artery; BA, basilar artery; VA (V4), intracranial vertebral artery segment 4; ICH, indicates intracerebral hemorrhage; mTICI, Modified treatment in cerebral infarction; SAH, subarachnoid hemorrhage.

### 3.3 Representative case

**Figure 4:**
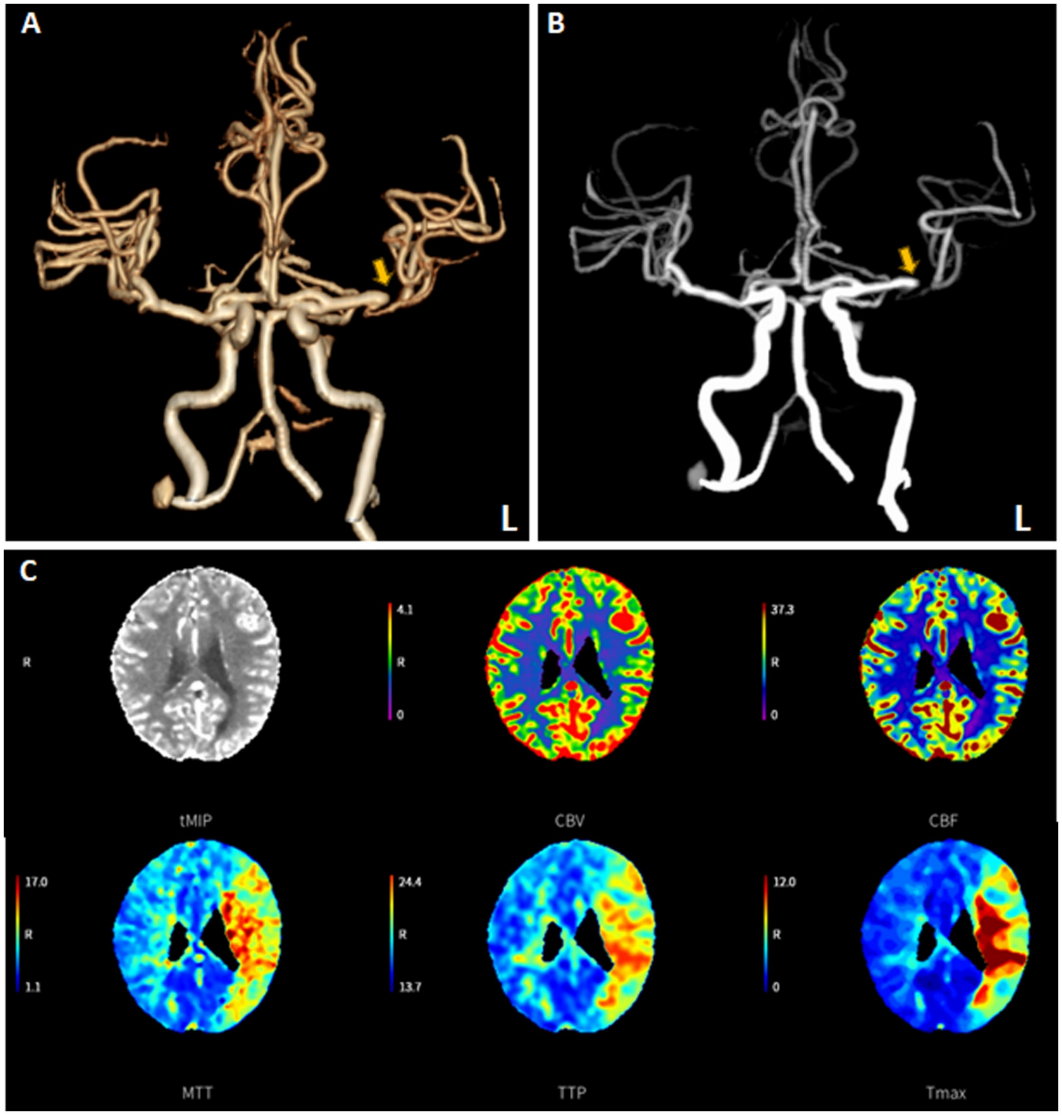
The “one-stop-shop” computed tomography (CT) images of an emergent admission patient presenting with a National Institutes of Health Stroke Scale of 21. A flat-detector CT scan was performed to exclude intracranial hemorrhage. CT angiography (CTA) demonstrated the distal M1 occlusion of the left middle cerebral artery **(A,B)**. CT perfusion (CTP) imaging revealed the presence of perfusion mismatch at the corresponding cerebral tissue **(C)**.

An 82-year-old female patient presented to the emergency room three hours after experiencing sudden onset aphasia and right-sided paralysis. The patient’s NIHSS score was 21. CTA showed an occlusion of the M1 segment of the middle cerebral artery (MCA) (Figures 4A,B), while CT perfusion showed a delayed time to peak in the MCA territory (Figure 4C). The patient was diagnosed with an acute ischemic stroke due to cerebral large vessel occlusion. Given the absence of contraindications, intravenous rt-PA was administered 45 min after arrival at the hospital, followed by bridging therapy.

**Figure 5:**
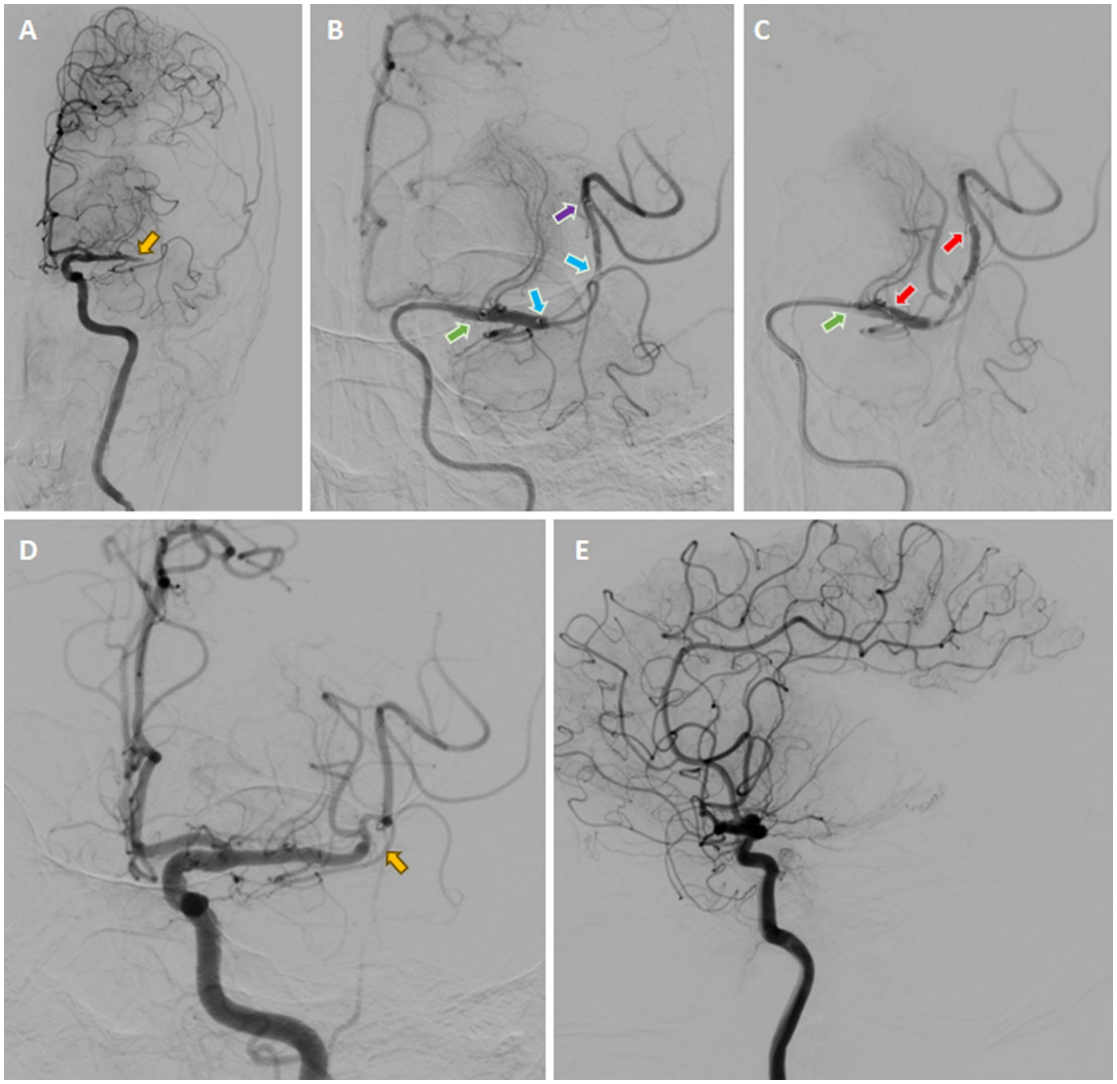
Digital Subtraction Cerebral Angiography (DSA) demonstrated the presence of an occlusion in the left M1 segment of the MCA (yellow arrow) **(A)**. Once the microcatheter had been navigated through the occluded segment, contrast was injected from both the aspiration catheter (green arrow) and the microcatheter (purple arrow), a technique known as the dual-channels bolus contrast injection (Dc-BCI). This revealed the total length of the thrombus (between two blue arrows) **(B)**. Subsequently, a stent retriever (illustrated by the two red arrows) was deployed over the thrombus **(C)**, resulting in an immediate bypass effect at the occluded artery. Subsequently, the SWIM technique (Solitaire stent retriever in combination with the intracranial support catheter aspiration for mechanical thrombectomy) was performed. The angiography results demonstrated reperfusion of the superior division M2, while the inferior division M2 remained occluded (yellow arrow) **(D, E)**. *Yellow arrow: site of occlusion; Green arrow: tip of aspiration catheter; Purple arrow: tip of microcatheter; Blue arrows: proximal and distal ends of embolus (also known as total thrombus length); Red arrows: proximal and distal ends of stent (also known as total stent length).

**Figure 6:**
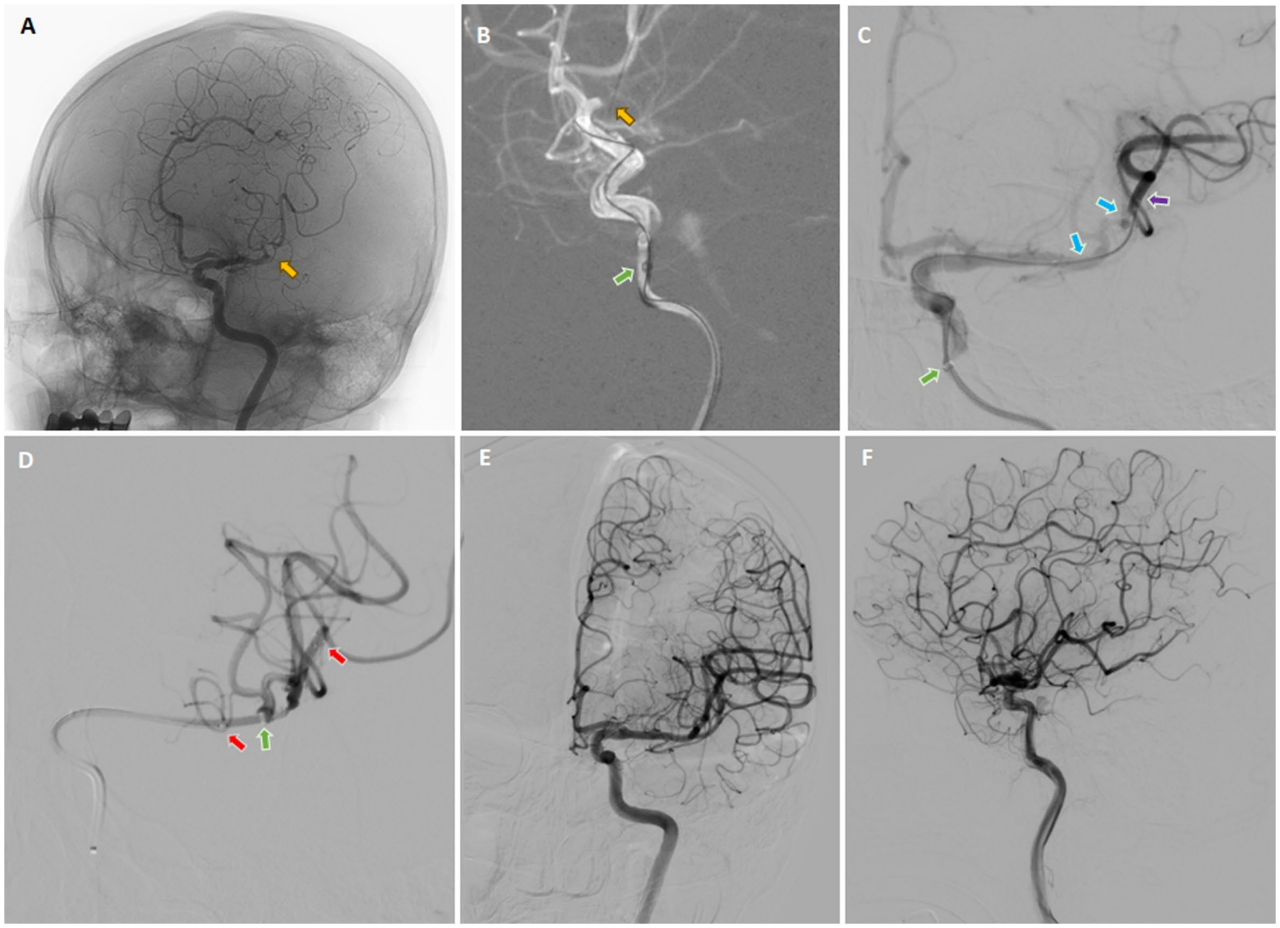
Digital subtraction cerebral angiography (DSA) in three dimensions was employed to confirm the occlusion of the inferior division M2 (yellow arrow) **(A)**. On the working angle, the roadmap provided a clear view of the occlusion site (yellow arrow) **(B)**, which allowed the microcatheter to pass through the occluded segment. Subsequently, contrast was injected from both the aspiration catheter (green arrow) and the microcatheter (purple arrow), a technique known as the dual-channels bolus contrast injection (Dc-BCI), in order to reveal the total thrombus length (between two blue arrows) **(C)**. Subsequently, a stent retriever (between two red arrows) was deployed over the thrombus **(C)** and the aspiration catheter was ascended forward to make direct contact aspiration (green arrow) **(D)**. The SWIM technique (Solitaire stent retriever in combination with the intracranial support catheter aspiration for mechanical thrombectomy) was then performed. The angiography confirmed complete reperfusion **(E,F)**. *Yellow arrow: site of occlusion; Green arrow: tip of aspiration catheter; Purple arrow: tip of microcatheter; Blue arrows: proximal and distal ends of embolus (also known as total thrombus length); Red arrows: proximal and distal ends of stent (also known as total stent length).

The 8-F guiding catheter was positioned in the proximal cervical ICA and the 5-F aspiration catheter was coaxially advanced to the distal C1 segment of the ICA (Figure 5A). The 3D rotational angiography was performed through the aspiration catheter, confirming occlusion of the left M1 segment of the MCA. After the C-arm arrived at the best working angle, the Rebar-18 Microcatheter (Medtronic, Minneapolis, MN, USA) was slowly navigated through the occluded segment of M1 and arrived at the M2 over the Synchro® microguidewire. After confirming that the microcatheter had arrived at the true arterial lumen, the Dc-BCI technique was used to precisely reveal the size of the thrombus (Figure 5B). Subsequently, a 5 × 22 mm Embotrap™ stent retriever was then delivered through the microcatheter and deployed over the thrombus (Figure 5C), resulting in an immediate bypass effect at the occluded artery. The stent retriever was deployed for 5 min, followed by retrieving into the aspiration catheter under constant aspiration. The angiography confirmed the reperfusion of the superior division M2 (Figures 5D,E). However, the inferior division M2 remained occluded. The same recanalization strategy was performed (Figures 6A–C). After the Dc-BCI, the 5 × 22 mm Embotrap™ stent was partially deployed over the occlusion, and the bypass effect was again observed (Figure 6D). The stent was recaptured and withdrawn 5 min later. Post-stent arteriography demonstrated a successful reperfusion of the right MCA (Figures 6E,F). XperCT showed no signs of procedural complications. The patient exhibited recovery of language function and muscle strength to level II on the second post-surgical day (NIHSS score 8).

## 4 Discussion

EMT has become the standard of care for cases of acute ischemic stroke induced by large vessel occlusion. The success of EMT depends on three factors, which are interventional technique- (6, 7), thrombus- (27) or stent-related (15). Previous studies have highlighted the importance of obtaining imaging characteristics of occlusive thrombi as this can provide valuable prognostic information for EMT (15). It is therefore essential to ascertain the precise length and diameter of a thrombus in order to maximize interaction between the thrombus and stent-retriever, thus achieving successful immediate recanalization. This study presents a technique for accurately identifying the size information of the thrombus (proximal and distal location and diameter), which has been demonstrated to enhance the efficacy of EMT.

The physical interaction between the retriever stent and the thrombus is of paramount importance in the successful implementation of EMT. This necessitates the use of an optimal retriever stent size and precise positioning. Nevertheless, these issues are currently unresolvable. Firstly, with regard to the selection of stent retriever size, there is currently no guidance available. Although one study suggested otherwise (28), the majority of studies support the idea that longer stent retrievers could enhance EMT performance in acute stroke. This is because stent size is positively correlated with a higher rate of successful first-pass and mTICI 2b/3 reperfusion. For example, Haussen et al. (8) and Zaidat et al. (9) reached the same conclusion that the longer stent retriever is an independent predictor of successful reperfusion, as the longer stent retriever demonstrated the highest rate of first-pass reperfusion compared to those with larger diameter or shorter length. This result was consistently presented in the subsequent studies (29–31). The rationale behind this conclusion is that longer retrievers have a greater surface area for interacting with the thrombus, thereby ensuring higher radial forces along the clot during retraction and reducing the possibility of thrombus retention. While this hypothesis may be plausible, it is important to note that EMT can cause severe damage to the vessel walls, such as endothelial denudation, thrombus deposition, thickening of the internal elastic lamina, and degeneration of the elastic fibers (16–18). Specifically, this vascular injury is more likely to happen in EMT using a stent retriever as compared to the aspiration technique (16, 18). Thus, while a longer stent retriever may lead to better clinical outcomes, it can also cause more severe damage to cerebral arteries, potentially leading to EMT failure due to new embolism events resulting from vascular injury. Based on these facts, we tend to favor the hypothesis proposed by Belachew et al. (15) that the ratio of thrombus-length to stent-retriever-length is more valuable than thrombus-length or stent-retriever-length alone in predicting favorable EMT and clinical outcomes. In the current study, since the thrombus length could be clearly identified by the Dc-BCI technique, an appropriate stent retriever could be chosen to reach a favorable thrombus-length/stent-retriever-length ratio. Furthermore, the positioning of the stent retriever is a critical element that affects the results of EMT procedure (32). It is not sufficient for the stent retriever to merely cover the entire thrombus. Rather, the optimal positioning requirements for recanalization vary across different brands of stent retrievers. For example, the Solitaire stent-retriever is advised to be positioned in a central location within the clot (33) while the proximal end of the first element of the EmboTrap™ should be placed at the beginning of the clot (34). In the absence of precise positioning of the distal end of the thrombus during EMT, the optimal stent-retriever positioning may be difficult to ascertain, potentially impacting the final outcome of recanalization. By employing the Dc-BCI technique, the stent retriever can be positioned in the optimal location with respect to the thrombus, thereby achieving a secure engagement between the thrombus and the stent retriever. In the present study, we achieved a favorable recanalization rate, which we attribute not only to the surgical technique and instruments, but also to the Dc-BCI technique, due to a number of advantages, as outlined below. Primarily, the stent retriever is capable of entrapping the thrombus in its entirety, thereby ensuring the most extensive integration surface and uniform distribution of radial forces along the clot during retraction. Secondly, a stent retriever (especially for Embotrap™) of appropriate length could be placed distally to the clot in order to capture clot fragments or protrusions through the stent. This may serve to mitigate the risk of dislodgement of clot fragments and the occurrence of secondary downstream embolic events. Thirdly, the use of an appropriate stent length or partial stent deployment may reduce the risk of vascular injury and preserve the physiological function of the cerebral artery to the fullest extent.

Currently, several imaging modalities are available for the purpose of identifying the size and location of thrombi, thereby assisting clinicians in the selection of optimal treatment candidates. The non-contrast CT revealed that the hyperdense vessel signs can be determined as the predictor of thrombosis. However, this method could only provide an approximate length of occlusion (20). The CTA remains the cornerstone among the acute ischemic stroke workflow due to its high accuracy in presenting cerebral arterial vasculature, near-universal availability, and fast turnaround times. However, CTA is unable to provide thrombus length in up to one-third of the stroke patients (21), primarily due to inadequate distal flow from the collaterals to a major occlusion and thus affect the identification of the thrombus’s distal location (20). Furthermore, CTA is also a highly time-consuming process (19) and regularly overestimates thrombus length as the distal end of the thrombus is not delineated (35). Past literature favored MR-based thrombolysis over CTA due to the advantages of MR in the assessment of acute stroke. Specifically, the diffusion-weighted imaging (DWI) can predict infarct core and the presence of hemorrhage, while the T2- (22, 23) and susceptibility-weighted sequence (24) can provide vital information regarding the thrombi’s location and length. However, the high potential demand for emergent MR scanning for stroke evaluation is in limited usage due to availability, patient comorbidities, and cost-effectiveness. Duplex ultrasonography (DUS) is also a useful imaging modality for stroke assessment, which can provide information about both emboli characteristics and the composition of the arterial wall. However, its usage is limited in extracranial carotid artery segments. Meanwhile, transcranial Doppler lacks an approved diagnostic protocol for cerebral artery and can only provide indirect evidence (e.g., absent or asymmetry of blood flow velocity) to diagnose stroke (36). Beyond that, the majority of these modalities are constrained to specific application scenarios, namely the perioperative period. Recently, Hofmeister et al. (37) reported that intraoperative contrast-enhanced cone beam CT (CE-CBCT) during EMT could markedly enhance the visualization of the distal edge of the clot during EMT. However, the cost of CE-CBCT might impede its utilization in numerous medical facilities. Thus, there is currently no effective and convenient method to predict thrombus characteristics, especially the size, during daily clinical practice. In the current study, we introduce a technique that could precisely identify thrombus length during EMT. As mentioned in the previous paragraph, understanding the thrombus size, especially the length, can help clinicians select an appropriate stent retriever, which prompts successful recanalization. Therefore, we believe that our technique will have a positive impact on clinical EMT practice.

This study has some limitations. Firstly, it did not evaluate the characteristics of thrombus composition in detail, which may also influence recanalization success. Secondly, stent retriever selection was at the discretion of the neuro-interventionalists, allowing for possible selection bias. It is also uncertain whether longer stents would have been equally safe and efficient in cases where some thrombi exceeded the longest stents available. Furthermore, the research team was not blinded to the clinical presentation, which could introduce bias to the results. Additionally, the study is limited by a small sample size from a single institution and the absence of a control group. Finally, during Dc-BCI, up to 10 mL of contrast was injected via the aspiration catheter. This has the potential to raise concerns due to the risk of local pressurization of the arterial bed. However, the absence of complications related to contrast injection in this study suggests that the procedure is safe. This assertion is supported by the following argument. Firstly, the precise amount of contrast material injected was less than 10 mL. This is attributable to the fact that the inner volume of the aspiration catheter sacrifices part of the injected contrast. Furthermore, it has been observed during clinical practice that residual contrast agent is invariably present in the syringe following the cessation of Dc-BCI, as the procedure is immediately terminated once an angiogram attains a satisfactory quality. In addition, since the Dc-BCI was performed via manual injection, the surgeon may have experienced direct force feedback during the injection procedure. This feedback could have allowed the surgeon to adjust the injection power to just counteract the blood pressure, but not to overexert it. Consequently, this approach may have led to a substantial decrease in pressure applied to the arterial bed compared to that of automated contrast injectors.

In conclusion, this study presents a novel technique for EMT, designated as Dc-BCI, which is capable of precisely identifying the size and location of thrombi. In both laboratory studies and clinical research, we demonstrated that the Dc-BCI could effectively reveal size information about the thrombus, thereby achieving optimal recanalization results. Therefore, we believe that the Dc-BCI has the potential to enhance the outcomes of EMT, as it helps to optimize the interaction between the stent retriever and the thrombus, while simultaneously reduce the risk of vascular injury that is associated with the prolonged use of the stent retriever.

## Data Availability

The datasets presented in this article are not readily available due to the ongoing nature of clinical trials. Requests to access the datasets should be directed to the corresponding author.
